# The Glucose-Regulated *MiR-483-3p* Influences Key Signaling Pathways in Cancer

**DOI:** 10.3390/cancers10060181

**Published:** 2018-06-04

**Authors:** Felice Pepe, Rosa Visone, Angelo Veronese

**Affiliations:** 1 Comprehensive Cancer Center, The Ohio State University, Columbus, OH 43210, USA; Felice.Pepe@osumc.edu; 2 Ageing Research Center and Translational Medicine-CeSI-MeT, 66100 Chieti, Italy; r.visone@unich.it; 3 Department of Medical, Oral and Biotechnological Sciences, G. d’Annunzio University Chieti-Pescara, 66100 Chieti, Italy

**Keywords:** *miR-483-3p*, metabolism, *TP53*, CTNNB1, chemoresistance, cancer, *miR-145-5p*

## Abstract

The *hsa-mir-483* gene, located within the *IGF2* locus, transcribes for two mature microRNAs, *miR-483-5p* and *miR-483-3p*. This gene, whose regulation is mediated by the the CTNNB1/USF1 complex, shows an independent expression from its host gene *IGF2*. The *miR-483-3p* affects the Wnt/β-catenin, the TGF-β, and the TP53 signaling pathways by targeting several genes as *CTNNB1*, *SMAD4*, *IGF1*, and *BBC3*. Accordingly, *miR-483-3p* is associated with various tissues specific physiological properties as insulin and melanin production, as well as with cellular physiological functions such as wounding, differentiation, proliferation, and survival. Deregulation of *miR-483-3p* is observed in different types of cancer, and its overexpression can inhibit the pro-apoptotic pathway induced by the TP53 target effectors. As a result, the oncogenic characteristics of *miR-483-3p* are linked to the effect of some of the most relevant cancer-related genes, *TP53* and *CTNNB1*, as well as to one of the most important cancer hallmark: the aberrant glucose metabolism of tumor cells. In this review, we summarize the recent findings regarding the *miR-483-3p*, to elucidate its functional role in physiological and pathological contexts, focusing overall on its involvement in cancer and in the TP53 pathway.

## 1. Introduction

MicroRNAs (miRNAs) are noncoding RNAs of about 18–28 ribonucleotide lengths, which modulate gene expression by inhibiting their translation or promoting their mRNA degradation [1]. MicroRNAs play a critical function in the homeostasis of central cellular processes, and their deregulation in human neoplasm has mainly been proven [2,3,4].

**Figure 1:**
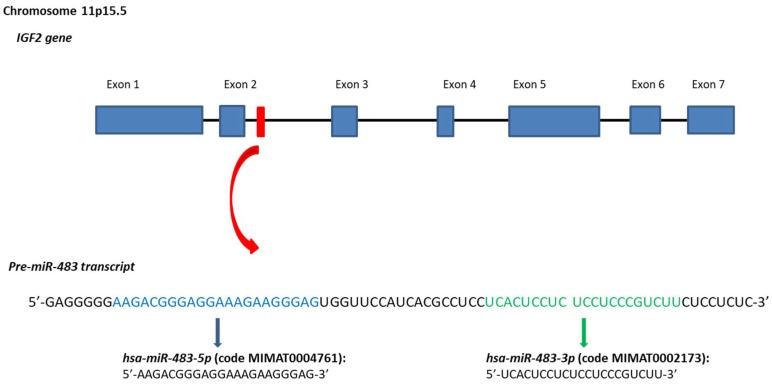
Stem-loop sequence of the *hsa-miR-483* and mature miRNAs. In the Figure are reported genomic position, sequence of the *hsa-miR-483* gene. Sequences data from miRBase database [27,28,29,30,31].

The *hsa-miR-483* gene is a mammal-conserved microRNA that resides at the 2nd intron of the human *insulin growth factor 2* (*IGF2*) gene at the *11p15.5* chromosome region [5] (Figure 1). The genomic localization of this microRNA is of particular interest. Indeed, the *IGF2* is an imprinted gene, expressed by the paternal allele to produce an important fetal insulin growth factor. Defects in the imprinting of the *IGF2* locus are observed in the Beckwith-Wiedemann syndrome, which increases the incidence of pediatric malignancies as nephroblastoma (Wilms’ tumor), hepatoblastoma, and rhabdomyosarcoma [6]. Moreover, also adult tumors are linked to genetic and epigenetic defects of this imprinted locus such as colorectal cancer (CRC), hepatocellular carcinoma (HCC) and breast cancer (BrCA) [7,8] pointing out to *IGF2* as the main oncogene of this genomic locus. However, a transgenic mouse model for *IGF2* (without including the *miR-483* gene) exhibited several features associated with the Beckwith-Wiedemann syndrome without association to any neoplasia [9]. These data suggested the *IGF2/miR-483* locus as the oncogenic unit of the *11p15.5* chromosome region instead of the *IGF2* alone [10,11,12].

The *hsa-miR-483* gene encodes for two mature miRNAs: *miR-483-5p* and *miR-483-3p*. Both are found deregulated in different types of cancer. *MiR-483-5p* is abnormally observed in the serum of cancer patients suggesting its possible utilise as cancer biomarker in various malignancies [13,14,15,16,17,18,19,20,21,22,23,24,25,26]. On the other hand, the *miR-483-3p* is extensively studied concerning its function on different cancer-related pathways as chemoresistance, immune-evasion, cancer metabolism and resistance to apoptosis by targeting several genes (Table 1).

## 2. The Regulation of Transcription of *MiR-483-3p*

### 2.1. The IGF2/MiR-483 Locus

*MiR-483* maps at 11p15.5, a chromosomal region defined Multiple Tumor-Associated Chromosomes Region 1 (MTACR1). Genetic and epigenetic abnormalities in this chromosomal region have been associated with various human neoplasms and with the cancer-predisposing Beckwith-Wiedemann syndrome (BWS). In particular, genetic aberrations at 11p15 are typically detected in more than 80% of Wilms’ tumors [42] and, albeit, less frequently, in other more common adult neoplasms [43,44,45,46].

The *miR-483-3p* host gene, *IGF2*, is part of the *IGF2*/*H19* genetic locus. This locus is one of a few hundred imprinted DNA regions in mammals and one of the best studied in human. *IGF2* is expressed exclusively by the paternal allele whereas the *H19* gene is maternally expressed. This allelic specific transcription is dictated by the methylation status of the *IGF2/H19* Imprinted Control Region (ICR), a DNA sequence located between these two genes. At the paternal allele, the ICR is methylated whereas in the maternal allele is de-methylated [47]. In cancer, especially in Wilms’ tumor, aberrant methylation of the ICR causes the reactivation of the maternal silent *IGF2* allele and the silencing of the active *H19* maternal allele [8,48,49,50,51]. Nevertheless, *IGF2* murine transgenic models have highlighted features close to the BWS without tumor predisposition [9]. On the other hand, the Wilms’ tumor mouse model published by Hu et al. was generated by somatic ablation of *Wt1* and constitutional bi-allelic expression of the *IGF2* entire locus which contain the *mmu-miR-483* gene [52].

We observed that *miR-483-3p* is over-expressed in 100% of Wilms’ tumors, and that colorectal, breast and liver cancers exhibit high levels of *miR-483-3p* in about the 30% of the cases [12]. It was found a positive correlation between the expression of *miR-483-3p* and *IGF2* in different types of cancers as HCC, CRC and Wilms’ tumor, suggesting a co-regulation with *IGF2*. However, HCC samples exhibited the lower coefficient of correlation, several samples showed divergent expression between *IGF2* and *miR-483-3p*. Indeed, it was observed that *miR-483-3p* could be finely regulated in the HCC cells independently from its host gene *IGF2*. This independent mechanism of transcription involves DNA methylation, specific transcription factors, and cellular glucose concentration. Here we reported the last discoveries about the *miR-483-3p* regulation.

### 2.2. MiR-483-3p and β-Catenin

*MiR-483-3p* expression was found up-regulated in tumors (HCC, CRC, BrCA and Wilms’ tumor) [12] that show the β-catenin (CTNNB1) signaling frequently dysregulated [53,54,55]. Interestingly an association between the mutational status of genes related to β-catenin pathway (APC, CTNNB1, and AXIN1) and *miR-483-3p* expression, but not *IGF2*, was detected in HCC. These observations, together with in vitro experiments showed an exclusive regulation of the *miR-483-3p* expression under the control of β-catenin transcription factor [35].

However, *CTNNB1* itself is a target of *miR-483-3p*, triggering a negative regulatory loop. This regulatory mechanism becomes ineffective in cells harboring an activating mutation on the *CTNNB1* coding sequence (ser45 deletion). In pathological conditions, this mutation permits to the protein to evade the regulative loop and cause high expression of both elements.

**Figure 2:**
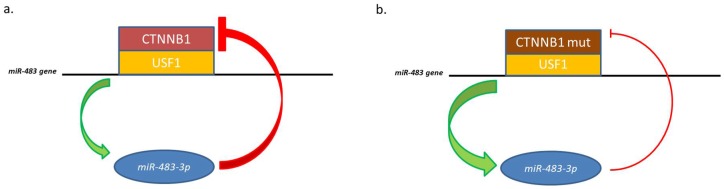
Representation of *miR-483-3p* feedback with the USF1/CTNNB1 complex. (**a**) Physiological negative feedback; (**b**) CTNNB1 mutated is no more regulated by *miR-483-3p*, which expression increase.

Analysis of the minimal promoter region of the *miR-483* located the β-catenin responsive element closely upstream the microRNA gene and identified it as an E-box motif (CACGTG). This E-box interacts with the basic helix-loop-helix protein upstream stimulatory transcription factor 1 (USF1) which was demonstrated be the co-factor of β-catenin to regulate the *miR-483* gene [35] (Figure 2).

### 2.3. MiR-483 and the Glucose Metabolism

The association of glucose metabolism to the regulation of *miR-483-3p* is supported by several studies that connect CTNNB1 and USF1 activity to cellular glucose metabolism [56,57,58] and by the fact that *miR-483-3p* maps at the INS-*IGF2* locus, which is involved in the insulin pathway [7,59]. Moreover, it was observed that the *miR-483-3p* is up-regulated in streptozotocin-induced diabetic mice, and cultured cardiomyocytes mimicking hyperglycemia [60]. Studies on HepG2 cells (Hepatoblastoma cell line) showed that the expression of *miR-483-3p* is significantly affected by the extracellular concentration of glucose [61]. Indeed, *MiR-483-3p* expression was reduced by both glucose starvation and treatment with the glucose antagonist 2-deoxy-D-glucose (2-DG). The treatment with 2-DG affected the levels of both *miR-483-3p* and its precursor *pri-miR-483* in a concentration-dependent way. Moreover, this regulation was independent of *IGF2* expression. Glucose concentration affects the *miR-483-3p* expression by the *O*-linked β-*N*-acetylglucosamine transferase (OGT). OGT protein mediates the addition of a *N*-acetylglucosamine in an *O*-glycosidic linkage (*O*-GlcNAcylation) to protein serine or threonine residues [62,63], and influences several cellular processes such as, RNA transcription [64], insulin signaling [65], Wnt/β-catenin signaling [66,67] and mouse embryonic development [68]. This post-translational modification is executed in the cytoplasm, using the activated derivative of the glucose metabolism Uridine diphosphate *N*-acetylglucosamine or UDP-GlcNAc as substrate, and it is commonly considered a cellular nutrient sensor and a metabolic regulator [69,70]. OGT contributes to β-catenin stabilization [66,67] and consequently to the regulation of *miR-483-3p* by the CTNNB1/USF1 complex at the E-box element of the *miR-483* promoter. Silencing OGT by siRNA or inhibitors as azaserine, reduces the *miR-483-3p* expression, and the affinity between USF1 and the E-box sequence. This suggests that OGT is fundamental for the CTNNB1/USF1 complex activity regulation of the *miR-483-3p* expression [61]. However, the authors do not exclude that OGT could influence the *miR-483-3p* expression also by other indirect mechanisms. Indeed, OGT regulates RNA transcription by *O*-GlcNAcylation of RNA polymerase II [71] and several critical transcription factors such as the bHLH transcription factor MYC proto-oncogene [72].

The decrease of *miR-483-3p* expression, by silencing OGT or by 2-DG treatment, was concomitant with the increasing of *BBC3* mRNA. These results suggest that the use of the glucose inhibitors could reactivate the TP53 pathway in those tumors where the over-expression of *miR-483-3p* blocks the apoptotic effector of *BBC3*/PUMA. However, despite promising in vitro results that show the 2-DG able to increase the efficacy of the chemotherapic drug 5-fluorouracil (5-FU); in vivo experiment on NSG mice transplanted with HepG2 cells, revealed that tumor cells with higher *miR-483-3p* expression were selected during tumor progression regardless the 5-FU/2-DG treatment [61].

### 2.4. MiR-483 and DNA Methylation

It was shown that a fragment of 151bp (IGF2-C_BI_), immediately upstream *miR-483* stem-loop, has strong insulator activity and binds to the CCCTC-binding factor (CTCF) [73]. CTCF is an important methyl-sensitive regulator of transcription involved in the epigenetic regulation of genomic imprinted loci by influencing genomic 3D conformation [74]. Accordingly, the methylation status of IGF2-C_BI_ correlates positively with the expression of *miR-483-3p* [35]. There are no direct data about the influence of the methylation status of the ICR of the *IGF2*/*H19* locus and the expression of the *miR-483* gene yet. However, the paternal allele expression, ruled by the ICR methylation, should reasonably open the chromatin also for the *miR-483* gene expression.

### 2.5. Other Mechanisms

Recently it was shown that the pseudogene HMGA1P7 could up-regulates *miR-483* through a competing endogenous RNA (ceRNA) mechanism that induces the Egr1 transcription factor, a positive regulator of *miR-483* expression [75].

It was also shown that the temperature could regulate the *miR-483* locus, so, a mild hypothermia induces an increased expression of the *miRNA* [76]. Moreover, *miR-483* may self-regulate its own expression independently of its host gene *IGF2* in human HeLa cells by activating the USF1 transcription factor [77].

## 3. Physiological Roles of *MiR-483-3p*

*MiR-483-3p* influences many biological processes. Here summary of the most known is provided:

### 3.1. Cell Cycle Regulation

In a study on skin wound repair, it was observed that *miR-483-3p* is accumulated in the terminal phase of the repair process and blocks the cell cycle of keratinocytes by targeting the phosphatase CDC25A. The *miR-483-3p* silencing leads to a continuous proliferation and delays the differentiation [34,78].

### 3.2. Melatonin Synthesis

In the pineal gland, it was observed a decreased expression of *miR-483-3p* during development, increasing of the *miR-483-3p* target *AANAT*, and melatonin synthesis [32].

### 3.3. Adipocytes Differentiation and Imprinting in Newborns

Administration of *miR-483-3p* modulates the capacity of adipocytes to differentiate and store lipids in vitro. These effects are probably mediated by translational repression of the growth/differentiation factor-3 (GDF3), a target of *miR-483-3p*. In vivo observations registered that *miR-483-3p* expression can be regulated by diet and can affect the development of the adipose tissues in off-springs. So, *miR-483-3p* is up-regulated in adipose tissue from low-birth-weight adult humans and pre-diabetic adult rats exposed to suboptimal nutrition in early life. The authors proposed that increased *miR-483-3p* expression causes lipotoxicity and insulin resistance by limiting storage of lipids in adipose tissue. In this view changes in miRNA expression during early-life nutrition could increase susceptibility to metabolic diseases affecting long-term health [79]. Finally, the authors observed, that in a cohort of 244 twins the environmental factors rather than genetics influence changes in *miR-483-3p* expression [80].

The effect of the diet on *miR-483-3p* regulation seems to act before birth. In mice, maternal high fat diet reduces the expression of a group of miRNAs in adult offspring liver, including the *mmu-miR-483-3p* [81]. These data attest the importance of the environmental factors on *miR-483-3p* regulation and their capacity to affect the cellular development and long-term health.

### 3.4. Matrix Production in Eye Cells

*MiR-483-3p* reduces the production of extracellular matrix (ECM) in Human Trabecular Meshwork Cells by targeting of SMAD4, and in turn reducing the activity of the TGF-β pathway in the eye. This relationship between *miR-483-3p* and the ECM production makes this microRNA a potential target to treat glaucoma [82].

### 3.5. Vascular Homeostasis

*MiR-483-3p* rules the vascular homeostasis controlling the components of the renin-angiotensin system by regulating four different genes of the renin-angiotensin system (RAS) [83]. Moreover, *miR-483-3p* is upregulated in endothelial progenitor cells (EPC) from deep vein thrombosis (DVT) patients and targets the serum response factor (SRF) to decrease EPCs migration and tube development, concomitantly to the increased rate of the apoptotic cells. In a rat model of venous thrombosis, *miR-483-3p* inhibition led to the improved ability of homing and thrombus resolution of EPCs [84].

### 3.6. Mesodermal Differentiation

As already mentioned, *miR-483-3p* could play an important function in differentiation. Mesoderm cells showed enrichment of *miR-483-3p* that was identified as a pivotal regulator for the differentiation to progenitor subpopulations toward lineage specification. *MiR-483-3p* effect was partially mimicked by repression of its target *PGAM1* gene [39]. 

## 4. *MiR-483-3p* in Cancer

### 4.1. MiR-483-3p Is Often Deregulated in Cancer

Since *miR-483-3p* is involved in numerous physiological processes, it is not surprising to find altered expression of this miRNA in diseases. *MiR-483-3p* was observed overexpressed in all the cases of cirrhotic liver and Wilms’ tumor studied [12]. Moreover, it was found upregulated in adrenocortical carcinoma [85,86], malignant mesothelioma [87], pancreatic ductal adenocarcinoma [41], and significantly overexpressed in large subsets of colorectal cancer [36], breast cancer and hepatocellular carcinoma [12]. Different authors observed that the *miR-483-3p* expression was increased in cancer cell lines from different tissues treated with chemicals with oncosuppressor activity or radiation. For example, *miR-483-3p* expression increased after treatment with cisplatin in ovarian cancer [40], selenium in adenocarcinoma [88], and 5-fluorouracil in HCC [61], or with radioactive stress in a lung cancer cell model [89]. Moreover, it was observed that lipotoxic substances in HepG2 cells induced deregulation of *miR-483-3p* [90]. Interestingly, the overexpression of *miR-483-3p* was associated with resistance to apoptosis and clone selection.

Colon organoid culture functionally confirmed the microRNA *miR-483* as a dominant driver oncogene at the *IGF2* locus; overexpression of *miR-483-3p* but not *IGF2* induced high-grade dysplasia of Apc-null organoids by increasing proliferation and invasion in vitro and tumorigenicity in vivo [11].

### 4.2. MiR-483-3p and the TP53/MiR145-5p Loop in Liver Cancer

Different mechanisms were proposed to explain how the *miR-483-3p* overexpression could drive advantages to cancer. For example, an important study showed how *miR-483-3p* can modulate the immune response against cancer cells. It was reported that *IGF1* and *IGF2* suppress their expression each other and their concentration can affect the immune response. *IGF1* promotes the development and cytotoxic activity of human NK cells [37], therefore the reduction of this protein by the augmented expression of *IGF2* by *miR-483-5p* [91], or by the direct targeting of *IGF1* by *miR-483-3p* [92,93], provide a protection to cancer cells by suppressing NK cells activity. 

Today, the most studied mechanism about how *miR-483-3p* could induce the chemoresistance in cancer cells involves the capacity of the *miR* to affect the pro-apoptotic TP53 signaling in the context of the liver cancer. In hepatocellular carcinoma (HCC), the expression of *miR-483-3p* is related to the mutational status of *TP53*, *CTNNB1* genes and impaired epigenetic mechanisms affecting the *IGF2/miR-483/H19* locus. Interesting, *CTNNB1* and *TP53* are the most commonly mutated gene HCC (in about the 50% of all cases), but mutations in these two genes are mutually excluding. Therefore, HCC tumors mutated in CTNNB1 seldom have mutations in TP53 [94]. Notably, *IGF2*, β-catenin and TP53 are related to cellular metabolism [95,96], and lipid and glucose metabolisms are impaired in HCC [97]. In this context, it was observed that the *miR-483-3p* acts as “onco-miR” targeting the pro-apoptotic gene TP53 Upregulated Modulator of Apoptosis protein (*BBC3*/PUMA) [12,35]. PUMA is one of the principal effectors of the TP53 apoptotic pathway, and reduction of PUMA can inhibit the apoptosis induced by the TP53 signaling activation. Therefore, *miR-483-3p* aberrant expression could be the elements that explain the mutual exclusion of the mutational status between CTNNB1 and TP53 in HCC. It was showed how mutations in CTNNB1, those permitting to escape the auto-regulation of the *miR-483-3p*, could lead to an accumulation of the protein and of *miR-483-3p*, blocking the pro-apoptotic activity of the TP53 pathway. In fact it was demonstrated that the overexpression of the *miR-483-3p* can overcome the *miR-145-5p*/TP53 axis in HepG2, and that the cellular glucose concentration has a major in the interplay between these factors [98].

**Figure 3:**
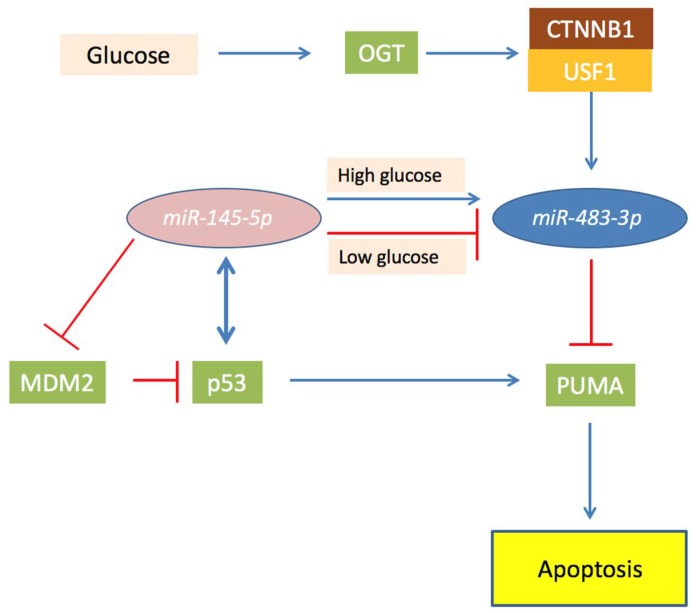
Schematic representation of the role of *O*-linked β-*N*-acetylglucosamine transferase (OGT) and *miR-483-3p* in the *miR-145-5p/TP53* axis in hepatocellular carcinoma (HCC). In the Figure are showed proteins (squares) and miRNAs (ovals) and their functional connection involved in the *miR-145-5p*/TP53 axis in HCC. Briefly, *miR-145-5p* targets MDM2 and reduces expression of *miR-483-3p*, permitting the activation of TP53 that induce PUMA and apoptosis. In case of OGT over-activity, *miR-145-5p* is unable to regulate *miR-483-3p* which expression is increased by CTNNB1 activity. This leads the *miR-483-3p* to target PUMA and blocking the pro-apoptotic effect of TP53.

*MiR-145-5p* induces p53-dependent apoptosis targeting MDM2 [99,100,101,102,103,104], an inhibitor of TP53 that induces its degradation by ubiquitination. Accordingly, *miR-145-5p* is generally down-regulated in several tumors, including hepatocellular carcinomas (HCCs) [105,106,107,108,109,110]. TP53 is a transcriptional activator of *miR-145-5p*, and it is also implicated in the microRNA maturation complex. This generates a positive pro-apoptotic feedback loop between *miR-145-5p* and TP53 [102,104,111,112]. However, notwithstanding the oncosupressor role of *miR-145-5p*, some HCC cases showed a physiological expression of this miRNA. Experiments on HepG2 cell line showed that ectopic constant activation of the TP53/*miR-145-5p* signaling induces apoptosis in almost all the cells, but additionally lead to the selection of clones that exhibit higher *miR-483-3p* expression. Depletion of *miR-483-3p* in these cells reinforces apoptosis, strengthening the concept that the overexpression of the *miR-483-3p* helps the cancer cells to overcome the programmed cell death. The glucose concentration has a critical role in the *miR-483-3p* and *miR-145-5p* connected functions. In liver cancer cells grown in low glucose conditions *miR-145-5p* lowered *miR-483-3p*, allowing apoptosis. Instead, when cells were grown in high glucose, the levels of *miR-483-3p* increased in response to *miR-145-5p*, reducing the apoptotic rate (Figure 3). Accordingly, *miR-145-5p* and *miR-483-3p* correlate negatively in the non-neoplastic livers, but positively in HCCs that have impaired glucose metabolism. This oncogenetic mechanism of the *miR-483-3p* could be common also in other types of tumors, but it has still to be directly proven. However, the connection between the TP53 pro-apoptotic pathway and *miR-483-3p* involves several factors that can have auto-regulative loops. For example, OGT activity was shown to upregulate *miR-483-3p* expression, and to potentially have an oncogenic role in cancer by glucose uptake, but at the same time, was shown that inhibition of OGA, the enzyme that catalyzes *O*-GlcNAc hydrolysis, stabilizes TP53 from degradation in cancer [113]. 

### 4.3. MiR-483-3p as Cancer Biomarker

Some authors evaluated the possibility to use the *miR-483-3p* as a biomarker for diagnosis and follow-up of cancer events [114]. *MiR-483* was observed overexpressed in esophageal squamous cell carcinoma (ESCC) tissues, and its overexpression was negatively correlated with the prognosis and positively correlated with multidrug resistance. In vitro functional experiments showed that *miR-483-3p* could promote the proliferation, migration and could inhibit cells’ sensitivity to chemotherapy drugs [115]. In patients with pancreatic ductal adenocarcinoma (PDAC) it was observed a high expression, relative to healthy controls of the *miR-483-3p* and *miR-21* in blood samples [116]. Moreover, some articles suggest that aberrant *miR-483-3p* expression is an early event in PDAC tumorigenesis and that is associated with tumor differentiation and prognosis, indicating *miR-483-3p* also as a potential therapeutic target for PDAC [117]. In addition, *miR-483-3p* overexpression in plasma from patients with adrenal cortical cancer and pancreatic ductal adenocarcinoma improved the diagnostic accuracy of these diseases [86,116], and some authors suggest the efficacy of the *miR-483-3p* as biomarker in prostate cancer as well [118]. In another study on colorectal cancer, was observed that in tumor tissues the expression of both *miR-483-3p*/-*5p* and *IGF2* was significantly up-regulated in more than 60% of cases with a concomitant positive correlation between the two genes. However, only *miR-483-5p* showed high levels in serum, useful as sensitivity biomarker for the CRC [36]. The *miR-483-5p* was found significantly up-regulated in the serum of a broad range of cancer and pathological conditions ascribing it as potential cancer biomarker [11,13,15,119,120,121,122,123,124].

### 4.4. Oncosuppressor Role of MiR 483-3p

Not all the observations in literature concord that *miR-483-3p* acts as an oncomiR. It was observed that the *miR-483-3p* expression is decreased in gastric, nasopharyngeal, and some cases of hepatocellular carcinomas [125,126,127,128]. *MiR-483-3p* sensitized squamous cell carcinoma cells to drug-induced apoptosis by the direct targeting of several anti-apoptotic genes, such as API5, BIRC5, and RAN [78]. In a microarray analysis of breast cancer samples, was observed that the *miR-483-3p* and *miR-483-5p* are significantly deregulated, and the authors underline that these miRNAs could have a key role during the initiation and the progression of the disease [129].

In the context of liver cancer, it was shown that *miR-483-3p* and *483-5p* are down-regulated in hepatitis B virus-associated HCCs [127]. Overexpression of *miR-483-3p* and -*5p* reduced liver fibrosis in murine model [130] In a study involving 18 HCC samples, the microRNAs *483-3p* and *-5p* were observed down-regulated in tumors [126]; *miR-483-3p* down-regulated in alcoholic hepatitis and in DDC (Diethyl 1,4-dehydro-2,4,6-trimethyl-3,5-pyridine-dicarboxylate) re-fed mice livers [33]. However, these studies do not consider the mutational status of the *TP53* or *CTNNB1* genes.

## 5. Discussion

In this work, we focused on *miR-483-3p* roles in cancer. It has been found involved in several aspects of the cellular homeostasis, and its ability to link crucial cellular pathways such as TP53, TGFβ, Wnt/β-catenin and metabolic signaling give to it a large impact on the regulation of neoplastic cells. Indeed, this short non-coding RNA exemplifies the exceptional role of the microRNAs linking epigenetics, metabolism, microenvironment, programmed cell death and cellular differentiation. Consequently, considering *miR-483-3p* as oncosuppressor or oncogene is challenging. However, in most of the cases reported in the literature, the overexpression of *miR-483-3p* is positively associated to cancer. There is another critical microRNA cancer-related that resemblance the *miR-483-3p*, *miR-221*. *Hsa-miR-221* is up-regulated in several types of cancer, directly induced by TP53 and able to targets several downstream effectors of the TP53 pathway as *BBC3*/PUMA, BMF, PTEN and MDM2 [131,132,133,134]. Therefore, the ability to downregulate downstream effectors of the TP53 pathway by microRNAs that in turn are regulated by TP53 seems to be a common feature at least in those tumors where these microRNAs are de-regulated.

For all these reasons the *miR-483-3p* represents a promising cancer therapeutic target. However, it is involved in pathways that are ruled by reversible changes that in turn control cellular plasticity. Since cancer cells exploit cellular plasticity to environmental adaptation and tumor progression [135], all the genetic, epigenetic and metabolic aspects related to *miR-483-3p* have to be unveiled in order to reach a productive targeting of this microRNA in an anti-cancer therapeutic view.

## Figures and Tables

**Table 1:** *miR-483-3p* verified targets. (h), (r) and (m) indicate respectively human, rat and murine tissues.

Gene	Tissues	Cells/Cell Lines	References
*AANAT*	Pineal gland (h), (r)	Neonatal pinealocytes (r), HEK293	[32]
*BBC3PUMA*	Kidney, Colon, Liver (h)	HEK293, HCT116, HepG2	[12]
*BRCA1*	Liver (m)		[33]
*CDC25A*	Keratinocytes (h)		[34]
*CTNNB1*	Kidney (h), Colon (h)	HEK293, HCT116	[35]
*DLC1*	Colon (h)	HCT116, SW480	[36]
*IGF1*	Natural Killer cells (h), Cardiomyocites (m)		[37]
*MK2*	Keratinocyte (h)		[38]
*MKI67*	Keratinocyte (h)		[38]
*PGAM1*	Kidney (h)		[39]
*PRKCA*	Ovarian (h)	IGROV-1	[40]
*SMAD4*	Pancreas (h)	SW1990, PANC1	[41]
*YAP1*	Keratinocyte (h)		[38]
